# Multiple Sclerosis and Demyelinating Disorders: Past, Present, and Future

**DOI:** 10.3390/jcm13185621

**Published:** 2024-09-22

**Authors:** Christos Bakirtzis, Maria Elephteria Evangelopoulos, Nikolaos Grigoriadis

**Affiliations:** 1 Multiple Sclerosis Center, Second Department of Neurology, School of Medicine, Aristotle University of Thessaloniki, 546 21 Thessaloniki, Greece; ngrigoriadis@auth.gr; 2 First Department of Neurology, Aeginition Hospital, National and Kapodistrian University of Athens, 115 28 Athens, Greece; evangelopme@med.uoa.gr

In the past two decades, there has been a considerable increase of our knowledge with regards to the pathophysiology and management of various demyelinating diseases of the central nervous system. Multiple sclerosis (MS), the most common amongst these disease entities, is now attributed to insidious neurodegenerative and neuroinflammatory processes. The management of people with MS (PwMS) has been substantially improved. In particular, diagnostic criteria have been refined [1], while preclinical conditions such as radiologically isolated syndrome, have been extensively studied [2,3]. Additionally, recent advances in therapeutic interventions have improved the disease outcomes [4]. Current research explores the feasibility of advanced neuroimaging techniques [5] and blood biomarkers such as the levels of neurofilament light chains [6], in clinical practice. Future perspectives include the implementation of digital monitoring tools in routine practice, thus enabling early detection and prevention of disease worsening [7]. Nevertheless, there is still need for further research in this field, in order to improve our understanding about the underlying mechanisms of the disease progression and of the inhibition of repairing processes. In light of the above, with this special issue, we aim to provide information about advances in phenotyping PwMS, personalization of treatment and long-term monitoring of MS.

Focusing on the near past COVID-19 pandemic era, our study group aimed to explore whether SARS-CoV-2 immunization improved COVID-19 disease outcome in people with MS [Contribution 1]. A Greek nationwide administrative database was analyzed and according to the study results, vaccination provided protection from hospitalization, but did not affect mortality. Factors associated with increased probability of hospitalization were older age, male sex and the presence of comorbid conditions. This study did not examine the effect of SARS-CoV-2 immunization in the MS disease course. However, in this special issue, Frahm et al., performed a prospective study, using digital patient reported data in the German MS population, in order to explore the effect of the vaccination in the incidence of MS relapses [Contribution 2]. This study compared annualized relapse rates after vaccination with those observed in various German cohorts and demonstrated that there was no increased short-term disease activity after SARS-CoV-2 immunization.

Current research in MS focuses in the progression of disability, it’s relation with various neurodegenerative processes and with the activation of cells of the innate immunity [8]. Some of the underlying mechanisms of neurodegeneration, seem to be shared among aging, MS and classic neurodegenerative diseases such as Alzheimer’s and Parkinson’s. The current evidence of these common processes at the molecular and cellular level are reviewed by Kesidou et al. [Contribution 3]. The authors additionally present the current knowledge about the impact of ageing processes in the healthy brain and in various diseases such as MS. The potential contribution of various clinical and environmental factors, to the accumulation of disability in people with MS, was investigated by Barcutean et al., in a large sample in Romania [Contribution 4]. The key finding of this study was that timely treatment with DMTs, even with those with moderate efficacy, has a positive impact in long term disability outcomes. The genetic component of MS pathophysiology is up to now partially unexplored [9]. Although some genes that raise MS susceptibility have been detected [10], further studies are needed in order to understand the contribution of the genetic component to MS occurrence. In this special issue, Bruzaite et al., focused on the STAT4 gene and STAT4 protein levels in the sera of a sample of 200 people with MS and 200 healthy controls in Lithuania [Contribution 5]. According to this study results, the STAT4 genetic variant rs7601754 is related with MS susceptibility, while STAT4 serum levels are found lower in PwMS.

In some parts of the world, the diagnosis and management of MS related symptoms remain challenging, since diagnostic procedures and therapeutic agents are not widely available. In this special issue, Khan et al., have reviewed current molecular and cellular biomarkers of the disease with the aim to provide a roadmap of prioritization of their access from people living in India [Contribution 6]. Besides accessibility, there is still need for further studies in the clinical expression of the disease. Vagias et al., have explored visual processing and cognitive functions in early MS in Australia [Contribution 7]. According to this study, distinct phenotypes can be observed with regards to the cognitive visual processing interaction. Interestingly enough, these phenotypes are not captured by routine neuropsychological assessment, but do have an impact on the performance of an individual in information processing speed testing. Comorbid conditions may be underestimated in MS and may lead in a poorer quality of life [11]. Rościszewska-Żukowska et al., have investigated the occurrence and the characteristics of headache amongst PwMS in Poland [Contribution 8], and found that more than half of the study sample, suffered from a primary headache. Their findings highlight the need for a holistic approach in MS management, with the aim to improve health related quality of life of the PwMS. The use of advanced diagnostic tools may shed light into poorly understood processes of the disease. Schimdt et al., have studied hyperreflective foci, observed via optical coherence tomography, in the retina of PwMS in Denmark [Contribution 9]. These foci may represent immune cells, however more studies are needed in order to better describe their exact nature. Nevertheless, the authors observed higher concentrations of them in PwMS, especially in those with a prior optic neuritis. This finding may perhaps represent repairing mechanisms and worth further study. 

In summary, the studies and reviews included in this special issue, demonstrate the need of a timely, multidisciplinary management of MS, as well the need for further research on the insidious processes that may be responsible for the long-term disability accumulation of PwMS. Therefore, we look forward to reading and editing new submissions in the new volume of “Multiple Sclerosis and Demyelinating Disorders: Past, Present, and Future—2nd Edition”.
